# Supplementary material for: Rapid turnover of life-cycle-related genes in the brown algae
Source: Genome Biol. 2019 Feb 14;20:35. doi: 10.1186/s13059-019-1630-6 (PMC6374913; doi:10.1186/s13059-019-1630-6)
Supplement: Supplementary file 3 — Protein alignments, corresponding Gblocks html file and CDS sequences for the lineage conserved gametophyte-biased genes. (ZIP 2109 kb) [file 13059_2019_1630_MOESM3_ESM.zip › Con_GA_alignments/Ec-00_010940.aa_ali.fasta-gb.htm]

Ec-00\_010940.aa.fasta.trans.aa\_ali.fasta


## Gblocks 0.91b Results

Processed file: **Ec-00\_010940.aa.fasta.trans.aa\_ali.fasta**  
Number of sequences: **2**  
Alignment assumed to be: **Protein**  
New number of positions: **729** (selected positions are underlined in blue)

```
                         10        20        30        40        50        60
                 =========+=========+=========+=========+=========+=========+
Ec-00_010940     MVVLSRARSATARAQAGHTAPTNVGNNVVAS-MRNSKSQPMMFANS----HPHMMRGVSG
MSTRG.48583.1    MAVHLRPRSAEIRKPPKCTALLAAHHVAGNSRLRSSKSQPIAVTSGNIAFKSRSSRATHA
                 ####################                                        


                         70        80        90       100       110       120
                 =========+=========+=========+=========+=========+=========+
Ec-00_010940     QRPSSGRSPKHPSTTTRHGIKGATKALCWYRLPATGGWVPSARAGHTAVTVGTDMVVFGG
MSTRG.48583.1    PTSTATHNGAATANMARSAQKRKSKVLNWYRLPATGGWVPSARAGHAAVSIGAQVILFGG
                                 ############################################


                        130       140       150       160       170       180
                 =========+=========+=========+=========+=========+=========+
Ec-00_010940     VVNGERVNELASFDVRTRGWRRLDSRESPGNAQG---RTPPPIAMATVPAPRAYHVAWAF
MSTRG.48583.1    IVNGERGNELTSFDLHTRVWRRVTPKASIVDGPGQKSLTTAATAVAAVPGPRAFHVAWAF
                 ##################################    ######################


                        190       200       210       220       230       240
                 =========+=========+=========+=========+=========+=========+
Ec-00_010940     GNDVYVHGGEG-PTGVRTDTDDPDLDGVFGSVGRDLFAEEDGGAPLDPVRVVRGAGAGKG
MSTRG.48583.1    NGNVYIHGGEGQATGETGLVGNPDLDDVLGSVGRDPFAEEDGGAPVEAGRIATAANGRSA
                 ###########  ###############################################


                        250       260       270       280       290       300
                 =========+=========+=========+=========+=========+=========+
Ec-00_010940     AAASGHRVRRTG-----------------------PSCGVERRPSVSVLEDLWKLDFRTL
MSTRG.48583.1    GVVSWPSVRSVGNAKGGAIGRQIARIDGTQSPLRPASSGGERRPAVSVLEDLWKFDSCAL
                 ############                        ########################


                        310       320       330       340       350       360
                 =========+=========+=========+=========+=========+=========+
Ec-00_010940     RWERLSSRLAPLGRKGHTASVVPLRDRPCVLIFGGAPAGRRGLSNALYSVDLAMLSAGEG
MSTRG.48583.1    QWERLSSRLAPLGRKGHTASVVSLRSRPHVLIFGGAPAGRRGLSNALYSADLALLSRGEG
                 ############################################################


                        370       380       390       400       410       420
                 =========+=========+=========+=========+=========+=========+
Ec-00_010940     TWERHKPSGTAPAPRHGHSLTAVAGGKRLVLFGGKAENGDRLGDIQILEVGGGGSLSWAV
MSTRG.48583.1    VWERHRPSGIAPAPRQGHTLTALIGGKRLVLFGGVAEGGELLRDIHVLETDG-DVLSWSA
                 ####################################################   #####


                        430       440       450       460       470       480
                 =========+=========+=========+=========+=========+=========+
Ec-00_010940     VQRPVGDLPPARHGHSACEVPTSAGVGARISRGAGVLVFGGEETANEGPEIRSTDSHKMF
MSTRG.48583.1    VQAPVGDIPTPRHGHSACEVPVTAGYAAGVS-GGGVLVFGGEGRTKEGQEVRSTDSHEIF
                 ############################### ############################


                        490       500       510       520       530       540
                 =========+=========+=========+=========+=========+=========+
Ec-00_010940     LYDPKESRWQAVETGHAFPIGRHGHSMSSIAGWTPPSFHPDGTLTASELAPPSTATAGCE
MSTRG.48583.1    LYDLKESRWQNVATGHAFPIGRHGHSMSSIVGWTPPSFHPDGALTAAEQAPRANASLTWG
                 ############################################################


                        550       560       570       580       590       600
                 =========+=========+=========+=========+=========+=========+
Ec-00_010940     RNATGVTWAAAEKDRRVG---GSSGTSAGGREPATRSTVAP-PASSLPSEAGVLQWGEKA
MSTRG.48583.1    RKTATTPGQRHNNTVRVAEGGGGVGPGVGTRGGAIESPATTNSEGNIPSDAAVLQWGQKA
                 #                    ################          #############


                        610       620       630       640       650       660
                 =========+=========+=========+=========+=========+=========+
Ec-00_010940     PTCAVIFGGLNSMYVSPEVWMLPLRWGERTVPFFSPKSEPEPNLQRGDGGAIGSGGDADR
MSTRG.48583.1    PCCAVVFGGLNSMYVNPEMWVLPLRWVEKAVQLSPPPPEPE---TRQD-----EDGESGR
                 #########################################                   


                        670       680       690       700       710       720
                 =========+=========+=========+=========+=========+=========+
Ec-00_010940     GKRELGMSTSGRLKGGVATAAATAQGRTAASADGGRRVQGVGGATVNKI-G--TGDGAWR
MSTRG.48583.1    GNR-LGFAAVANIKTAGAVAAATARRRAAPVAVDRTGTRGAG-MVVNATGGNRPADDAWR
                     ######################################             #####


                        730       740       750       760       770       780
                 =========+=========+=========+=========+=========+=========+
Ec-00_010940     LFQAGSTGVENARVKHRRASLPGGFAGFAFDANRS-------NGEQPIHEIGVAELAGVV
MSTRG.48583.1    LFQAGGISMGARTVDHRRASLPGGLTAEAARRAQDSLHPYHLQQHQQNLRHETRENAGGE
                 #############################                ###############


                        790       800       810       820       830       840
                 =========+=========+=========+=========+=========+=========+
Ec-00_010940     TAGPGSDNNDH--NGMAEATAEIEAELHRLRKIAYVSEKKLSAEVATRMHLEETIKEMRE
MSTRG.48583.1    LHGAVEREVDHDPGGMPGTAAEIEGELIRLKRIAYVAEKKLSAEVCARMHLEETNQDMQR
                 ###########   ##############################################


                        850       860       870       880       890       900
                 =========+=========+=========+=========+=========+=========+
Ec-00_010940     ETNKLRFSKESSEIQLREESRRHWHEGQKAKVQCASLERLLAEAYELLNMVELQHHLQVK
MSTRG.48583.1    EMKELSSSKESAELELREEAKRQWHEGQKAKSRCASLERLLAEAYELINMMGLQHHLQVK
                 ############################################################


                        910       920       930       940       950       960
                 =========+=========+=========+=========+=========+=========+
Ec-00_010940     SLMPALYGDPRLNREIQDPGEQRGIDKNSLHGPGQWPPQHHQQPQHPLENGDGNHPGRAA
MSTRG.48583.1    SLMPG-------------------------------------------------------
                 ####                                                        


                        970       980       990      1000      1010
                 =========+=========+=========+=========+=========+===
Ec-00_010940     EGRRSSAATAGNEATEDAAARQFGEVSGRGGVEATVAVRIAGVLGAGGPLVAV
MSTRG.48583.1    -----------------------------------------------------
```

```
Parameters used
Minimum Number Of Sequences For A Conserved Position: 2
Minimum Number Of Sequences For A Flanking Position: 2
Maximum Number Of Contiguous Nonconserved Positions: 8
Minimum Length Of A Block: 10
Allowed Gap Positions: None
Use Similarity Matrices: Yes
```

```
Flank positions of the 13 selected block(s)
Flanks: [1  20]  [77  154]  [159  191]  [194  252]  [277  412]  [416  451]  [453  541]  [562  577]  [588  641]  [665  702]  [716  749]  [766  791]  [795  904]  

New number of positions in Ec-00_010940.aa.fasta.trans.aa_ali.fasta-gb:  729  (71% of the original 1013 positions)
```
